# Tonggyu-tang, a traditional Korean medicine, suppresses pro-inflammatory cytokine production through inhibition of MAPK and NF-κB activation in human mast cells and keratinocytes

**DOI:** 10.1186/s12906-017-1704-5

**Published:** 2017-03-31

**Authors:** Hyo In Kim, Se Hyang Hong, Jin Mo Ku, Sooyeon Kang, Tai Young Kim, Yong Cheol Shin, Seong-Gyu Ko

**Affiliations:** 1 Department of Science in Korean Medicine, Graduate School, Kyung Hee University, Kyungheedae-ro 26, Dongdaemun-gu, Seoul, 02447 Republic of Korea; 2 Department of Preventive Medicine, College of Korean Medicine, Kyung Hee University, Kyungheedae-ro 26, Dongdaemun-gu, Seoul, 02447 Republic of Korea; 3 Department of Science in Korean Medicine, College of Oriental Medicine, Kyung Hee University, Seoul, 130-701 Republic of Korea

**Keywords:** Tonggyu-tang, Anti-inflammation, Hmc-1, HaCaT, Mapk, NF-κB

## Abstract

**Background:**

Allergic diseases including allergic rhinitis, asthma, and atopic dermatitis are increasing worldwide. Common medications used to treat these inflammatory disorders are anti-histamines and corticosteroids, but they have their own limitations such as short duration and severe side effects. Thus, interest in complementary and alternative medicine is continually growing. Here, we investigate the anti-inflammatory mechanisms of Tonggyu-tang (TGT), a traditional Korean medicine that has been used to treat patients with allergic nasal disorders.

**Methods:**

We measured mRNA expressions and production of pro-inflammatory cytokines such as interleukin (IL)-4, IL-6, IL-8 and tumor necrosis factor alpha (TNF-α) by RT-PCR and ELISA assays in HMC-1 (human mast cell line-1) and HaCaT cells, immortalized human keratinocytes. Moreover, we evaluated the effect of TGT on two major inflammation-related pathways, mitogen activated protein kinase (MAPK) and NF-κB signaling pathway in these two cells.

**Results:**

Our results revealed that that TGT significantly reduced the expression and production of inflammatory cytokines such as IL-4, IL-6, IL-8, and TNF-α in the agonist-treated HMC-1 and HaCaT cells. We also found that TGT suppressed MAPK signaling pathway including extracellular signal-regulated kinase (ERK), p38 mitogen-activated protein kinase (p38), and c-Jun N-terminal kinase (JNK) as well as NF-κB pathway, which are known to regulate inflammatory cytokine expression.

**Conclusion:**

Taken together, our results demonstrate that TGT inhibits expression of pro-inflammatory cytokines by suppressing MAPK and NF-kB pathway in both mast cells and keratinocytes, suggesting the potential use of TGT in treating allergic inflammatory diseases.

**Electronic supplementary material:**

The online version of this article (doi:10.1186/s12906-017-1704-5) contains supplementary material, which is available to authorized users.

## Background

Allergic diseases are often caused by numerous inflammatory mediators such as histamine, chemokines, and cytokines from immune cells, which are affected by inflammatory cytokines from T cells and immunoglobulin E (IgE) from B cells [1, 2]. When the allergens enter the human body, phagocytes such as dendritic cells and macrophages process and present them to T cells, resulting in differentiation of naïve T cells into cytotoxic T (Tc) cells or helper T (Th) cells. While activated Tc cells kill infected cells, Th cells, especially Th2 cells, release interleukin (IL)-4, IL-6 and IL-10 to induce B cells to produce IgE, which in turn activates mast cells for histamine secretion [3, 4].

Current medications commonly used to relieve symptoms of allergic reactions are anti-histamines and corticosteroids, but they have their own limitations such as short duration and severe side effects [5, 6]. Thus, the use of complementary and alternative medicine to treat allergic diseases is largely gaining an interest. In traditional Korean Medicine, several herbal medicines have been used for the treatment of allergic inflammation. Especially, Tonggyu-tang (TGT) composed of 12 herbs (Table 1), similarly Pyeongwee-San [7], Biyeom-Tang [8], Hyeonggaeyeongyo-Tang [9] and So-Cheon-Ryon-Tang [10] have shown anti-inflammatory effects on patients with allergic nasal disorder.

**Table 1:** Components of TGT

Tonggyu-tang; TGT	Component ratio (%)	References
* Ledebouriella divaricata* Hiroe	6.46	
*Angelica koreanum* Kitagawa	6.46	
*Angelica tenuissima* Nakai	6.46	22
*Cimicifuga heracleifolia* Kom.	6.46	
*Pueraria thunbergiana* Benth.	6.46	23
Ligusticum wallichii var. officinale Yook.	4.82	
*Atractylodes lancea* DC.	9.69	
*Thuja orientalis*l.	12.92	24–26
*Ephedra sinica* Stapf.	3.23	27, 28, 37
*Zanthoxylum schinifolium* S.Z.	3.23	29
*Asarum sieboldii* var. *seoulense* Nakai	2.58	30, 38
*Glycyrrhiza glabra*	6.46	31–34, 35
*Astragalus membranaceus* var. *mongholicus* Bung	12.92	12, 13, 36, 39
*Xanthium strumarium* L.	6.46	14, 40
*Magnolia denudate* Desr.	2.94	15, 41
*Mentha arvensis* var. *piperascens* Makinv.	2.47	16, 42
Total	100	

TGT was first seen in Dongeuibogam, seventeenth-century textbook on Korean medicine, written by the famous royal physician, Heo Joon [11]. In this study, we modified ingredients of the original TGT by adding *Astragalus membranaceus* var. *mongholicus* Bung, *Xanthium strumarium* L., *Magnolia denudate* Desr. and *Mentha arvensis* var. *piperascens* Makinv, which are known to possess anti-inflammatory activities [12–16].

Although TGT has been commonly used for the treatment of allergic diseases, its underlying molecular mechanism of anti-inflammatory effect is unknown yet. Therefore, we hereby investigate the anti-inflammatory effect and the detailed molecular mechanism of TGT in HMC-1 (human mast cell line-1) and HaCaT cells which both participate in allergic disorders.

## Methods

### Chemicals and reagents

TGT was provided by Hanpoong pharmaceutical company (Jeonju, Korea) in a form of powder, which was dissolved in D.W. Phorbol 12-myristate 13-acetate (PMA), dimethyl sulfoxide (DMSO), ionomycin, lipopolysaccharide (LPS) and 3-[4,5-dimetylthiazol-2-yl]-2,5-diphenyltetrazoliumbromide (MTT) were purchased from Sigma-Aldrich Co. (St. Louis, MO, USA). Dulbecco’s phosphate-buffered saline (DPBS), Iscove’s Modified Dulbecco’s Medium (IMDM), Dulbecco’s Modified Eagle’s medium (DMEM), penicillin and streptomycin were obtained from WELGNE (Gyeongsan, Korea). 3-(4,5-dimethylthiazol-2-yl)-5-(3-carboxymethoxyphenyl)-2-(4-sulfophenyl)-2H–tetrazolium (MTT) was from Promega (Maddison, WI, USA). Fetal bovine serum (FBS) was obtained from GR scientific (Bedford, UK) and EZ-cytox was purchased from DoGEN (Seoul, Korea). Human mRNA primers (IL-4, IL-6, IL-8, IL-13, tumor necrosis factor alpha (TNF-α), glyceraldehyde 3-phosphate dehydrogenase (GAPDH) were purchased from Bioneer (Daejeon, Korea). Antibodies were obtained from Cell signaling Technology, Inc. (Danvers, MA, USA), and enzyme-linked immunosorbent assays (ELISA) antibodies were obtained from BD Biosciences (San Jose, CA, USA) and R&D Systems (Minneapolis, MN, USA).

### Cell culture and treatment

HMC-1 cells were grown in IMDM and HaCaT cells in DMEM, all supplied with 1% penicillin and streptomycin and 10% FBS, incubated at 37 °C, 5% CO_2_ and 95% humidity. HMC-1 cells were stimulated with 5 ng/ml horbol 12-myristate 13-acetate (PMA) (Sigma-Aldrich, St. Louis, MO, USA) plus 500 ng/ml ionomycin and HaCaT cells were stimulated with 1 μg/ml LPS. After stimulating cells, TGT was treated at various concentrations for 24 h.

### Cell viability measurement with MTS assay or MTT assay

HMC-1 cells HaCaT cells were treated with various concentrations of TGT (0, 10, 20, 50, 100, 200,500 and 1000 μg/ml) with or without 5 ng/ml PMA plus 500 ng/ml ionomycin or LPS 1 μg/ml After 24 h, cells were treated with MTS or MTT reagent solution for 1 h, then absorbance was measured at 490 nm or 540 nm using a microplate.

### RNA extraction and RT-PCR

Total RNA was extracted using an R&A blue™ Total RNA Extraction Kit (iNtRON Biotech, Korea). For measurement of RNA concentration, a Nanodrop 1000 (Thermo Fisher scientific, Waltham, MA, USA) was used. cDNA was prepared from 1 μg of total RNA using a cDNA synthesis kit (Takara Bio Inc., Kusatsu, Japan). 1 μl of cDNA were used for RT-PCR assays. The list of primers used in this study is shown in Table 2.

**Table 2:** Reverse-Transcriptase PCR primer sequences of oligonucleotide

Oligonucleotide		
GAPDH	F	GCT CTT CAC CAC CAT GGA GA
	R	CGC CCA TCA CGC CAC AGT TT
IL-4	F	TGC CTC CAA GAA CAC AAC TG
	R	CTC TGG TTG GCT TCC TTC AC
IL-6	F	AAC CTT CCA AAG ATG GCT GAA
	R	CAG GAA CTG GAT CAG GAC TTT
IL-8	F	TCA GTG CAT AAA GAC ATA CTCC
	R	TGG CAT CTT CAC TGA TTC TTG
TNF-α	F	TGA GCA CTG AAA GCA TGA TCC
	R	ATC ACT CCA AAG TGC AGC AG

### Cytokine release measurement with ELISA

Enzyme-linked immunosorbent assay (ELISA) was performed using kits from R&D systems (Minneapolis, MN, USA) and BD Biosciences (San Jose, CA, USA). Briefly, samples and cytokine standards were added in 96-well plates coated with coating buffer at 4 °C for overnight and After washing with 0.05% Tween-20 phosphate-buffered saline (PBST) and blocked plates using Assay diluent with antibodies of IL-4, IL-6, IL-8, TNF-α (BD Biosciences, San Jose, CA, USA) and IL-13 (R&D systems, Minneapolis, MN, USA), and incubated at 37 °C for 1 h. After the plates were washed, biotinylated antibodies were added and the plates were left in RT for additional 2 h. The plates were then washed, incubated with AP for 30 min at 37 °C. Then TBS substrate solution was added and absorbance was measured at 450 nm by a microplate reader.

### Western blot assay

Cells were harvested, washed with 1 ml DPBS and lysed with RIPA buffer (150 mM NaCl, 1% NP-40, 0.5% DOC, 0.1% SDS, 50 mM Tris (pH 8.0), 1 mM EDTA, 1 mM PMSF, 1 mM NaF, 1 mM Na_3_PO_4_, 1 μg /ml aprotinin, leupeptin, pepstatin) and incubated in ice for 20 min. Total cell lysates were then centrifuged at 13,000×*g* for 20 min at 4 °C to remove the insoluble materials. Next, the total concentration of extracted proteins was determined at 15 μg, and then separated by 10% sodium dodecyl sulfate-polyacrylamide gel electrophoresis (SDS-PAGE) and transferred onto nitrocellulose membranes. The membranes were blocked with 0.1% PBST supplied with 2% skimmed milk and 2% bovine serum albumin (BSA) (Sigma-Aldrich, St. Loius, MA, USA) for 1 h at RT. After 4 times of 15 min washing with PBST, the membranes were incubated with primary antibodies diluted at 1:1000 at 4 °C overnight. After incubation, the membranes were washed with PBS for 15 min, 4 times, and then incubated with secondary antibodies diluted at 1:4000 for 1 h at RT. The protein signals were developed by the ECL detection kit (DoGEN, Seoul, Korea).

### Statistical analysis

Results are expressed as the mean ± standard error (S.E.) of independent experiments, and statistical analyses were performed using ONEWAY ANOVA to determine differences between groups. All statistical analyses were performed using GraphPad Prism 5 (GraphPad Software, Inc., La Jolla, CA, USA). Values with ^*^ *p* < 0.05 and ^#^ *p* < 0.05 are considered to indicate statistical significance.

## Results

### Effect of TGT on expression and production of inflammatory cytokines in HMC-1 cells

**Fig. 1:**
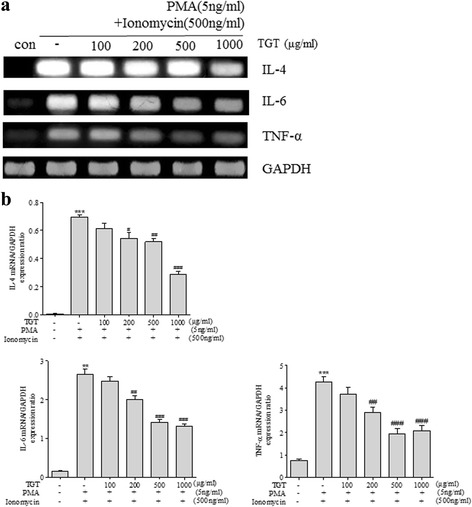
Effect of TGT on expression of pro-inflammatory cytokines in PI stimulated HMC-1 cells. **a** PI-stimulated HMC-1 cells were treated with or without different doses of TGT for 24 h. Cytokine mRNA levels were measured by RT-PCR. **b** The density of each band was calculated with image processing program, Image J, and represented as bar graphs. *; compared to control. #; compared to stimulated cells. Control; HMC-1 with PI (−), Stimulation; HMC-1 with PI (+)

**Fig. 2:**
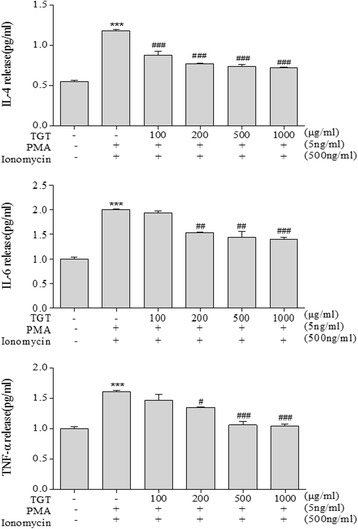
Effect of TGT on the release of pro-inflammatory cytokines in HMC-1 cells. PI-stimulated HMC-1 cells were treated with or without different doses of TGT for 24 h. The levels of secreted pro-inflammatory cytokines in the cell culture supernatant were measured by ELISA. *; compared to control. #; compared to stimulated cells. Control; HMC-1 with PI (−), Stimulation; HMC-1 with PI (+)

In order to examine the anti-inflammatory effects of TGT, we used the human mast cell line-1, HMC-1 cells, one of the most representative cells for studying inflammatory response. First, to evaluate the effect of TGT on HMC-1 cell viability, an MTS assay was performed. As a result, TGT did not affect HMC-1 viability up to concentration of 1000 μg/ml (Additional file 1: Fig. S1a). Next, to evaluate the anti-inflammatory effect of TGT, HMC-1 cells were stimulated with both PMA and ionomycin (PI), followed by treatment with various concentrations of TGT (100, 200, 500, 1000 μg/ml). As in Fig. 1a and b, PI treatment of HMC-1 cells increased mRNA levels of IL-4, IL-6 and TNF-α. Adding of TGT significantly reduced the mRNA expression of these cytokines. Note that in the PI-treated HMC-1 cells, TGT did not cause significant cell cytotoxicity up to 1000 μg/ml as well (Additional file 1: Fig. S1b). Furthermore, we assessed the effect of TGT on cytokine production by ELISA. As shown in Fig. 2, TGT treatment significantly reduced production of IL-4, IL-6 and TNF-α in the PI-treated HMC-1 cells.

### Effect of TGT on MAPK signaling pathway in HMC-1 cells

**Fig. 3:**
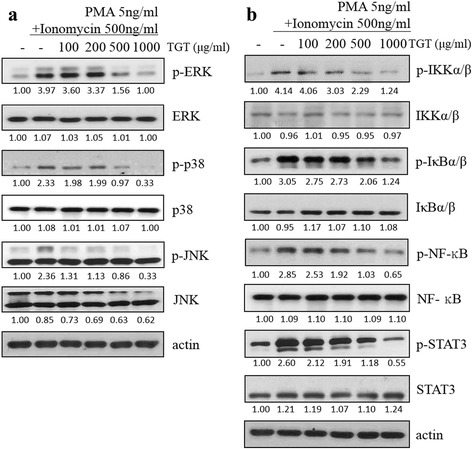
Effect of TGT on the protein expressions of MAPK and NF-κB signaling pathways in HMC-1 cells. PI-stimulated HMC-1 cells were treated with or without different doses of TGT for 24 h. The levels of protein expressions of (**a**) MAPK signaling pathway and (**b**) NF-κB signaling pathway in the whole cell lysates of HMC-1 cells were measured by western blot assays

To understand the molecular mechanism underlying the inhibitory effects of TGT on cytokine expression, we examined the mitogen activated protein kinase (MAPK) signaling pathway that is known to be closely related to allergy diseases by regulating the expressions of inflammatory cytokines such as TNF-α and IL-6 [17, 18]. We observed that TGT treatment of HMC-1 cells suppressed the PI-induced activation of extracellular signal-regulated kinase (ERK), p38 mitogen-activated protein kinase (p38), and c-Jun N-terminal kinase (JNK) in a dose-dependent manner (Fig. 3a).

### Effect of TGT on NF-κB signaling pathway in HMC-1 cells

NF-κB also plays a pivotal role in immune responses by regulating the expressions of various inflammatory cytokines. Under phosphorylated and degraded situations of IκB, NF-κB are translocated into the nucleus, then initiate inflammatory-related gene transcription [19, 20]. We found that increased phosphorylation levels of IKKα/β, IκBα/β, as well as STAT3 by PI treatment were inhibited by TGT co-treatment in a dose-dependent manner in HMC-1 cells (Fig. 3b).

### Effect of TGT on inflammatory cytokines in HaCaT cells

**Fig. 4:**
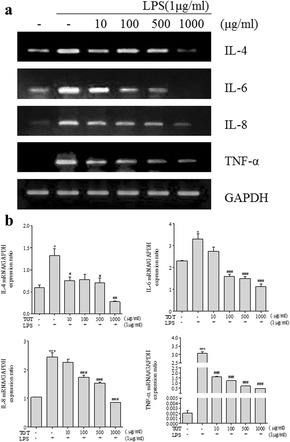
Effect of TGT on expression of pro-inflammatory cytokines in LPS stimulated HaCaT cells. **a** LPS-stimulated HaCaT cells were treated with or without different doses of TGT for 24 h. Cytokine mRNA levels were measured by RT-PCR. **b** The density of each band was calculated with image processing program, Image J, and represented as bar graph. *; compared to control. #; compared to stimulated cells. Control; HaCaT with LPS (−), Stimulation; HaCaT with LPS (+)

**Fig. 5:**
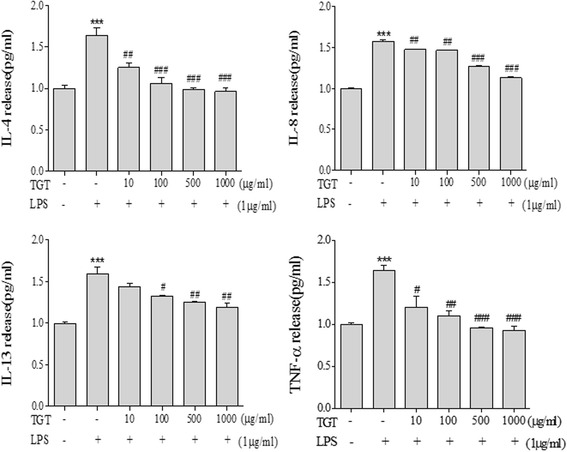
Effect of TGT on the release of pro-inflammatory cytokines in HaCaT cells. LPS-stimulated HaCaT cells were treated with or without different doses of TGT for 24 h. The levels of secreted pro-inflammatory cytokines in the cell culture supernatant were measured by ELISA. *; compared to control. #; compared to stimulated cells. Control; HaCaT with LPS (−), Stimulation; HaCaT with LPS (+)

Given that TGT suppresses the cytokine expression in HMC-1 cells, we next investigated the inhibitory effect of TGT on cytokine expression in HaCaT cells, an immortalized human keratinocytes. Of note, TGT did not show significant cytotoxicity in mock or LPS-treated HaCaT cells (Additional file 2: Fig. S2). Similar to HMC-1 cells, treatment of HaCaT cells with TGT markedly reduced the LPS-induced expression and production of inflammatory cytokines including IL-4, IL-6, IL-8 and TNF-α, as measured by RT-PCR and ELISA, respectively (Figs. 4 and 5).

## Discussion

Allergic diseases such as allergic rhinitis, atopic dermatitis and asthma share a number of pathogenic and epidemiological features. Although detailed mechanisms of these diseases at the cellular level are still unclear, recent studies have focused on the roles of immune cells, especially Th2 cells in the pathogenesis of these allergic disorders [21].

TGT is an herbal medicine composed of 12 different herbs that are frequently used for treatment of patients with nasal disorder [11]. In this study, we added 4 additional anti-inflammatory herbs including *Astragalus membranaceus*, *Xanthium strumarium*, *Magnolia denudate*, and *Mentha arvensis,* to the original constituents to increase TGT’s anti-allergic effects. Among these 16 herbs in the formulation of TGT, a number of studies have showed that extracts or an active compound from individual herbs have anti-inflammatory activities (Table 1) [12–16, 22–36]. Especially, six herbs including *Ephedra sinica* and *Asarum sieboldii* have been reported to have anti-histamine actions like inhibition of histamine release from mast cells or several histamine-mediated biological processes [37–42].

Since no studies have been performed to examine cellular and molecular actions of TGT, we sought to investigate the effects of TGT on human mast cells and keratinocytes. Mast cells play a key role in the development of allergic responses by releasing inflammatory mediators such as TNF-α, IL-6, IL-8, IL-13, and histamine [43, 44]. IL-6 stimulates the growths of neutrophils and B cells, and IL-13 influence the apoptosis and survival of eosinophils, respectively [45–47].

In this study, we showed that TGT significantly reduced the expression and production of inflammatory-cytokines in both HMC-1 and HaCaT cells. MAPK such as ERK, p38, and JNK are closely involved in the synthesis of inflammation mediators. Therefore, inhibitors targeting MAPKs are developed to reduce inflammation [48]. Our findings showed that TGT inhibits agonist-induced MAPK activation in a dose-dependent manner. Furthermore, we found that TGT treatment suppressed the agonist-induced activation of NF-κB pathway which is another important cellular signaling pathway for production of inflammatory cytokines. Overall, our results suggest that TGT can be effective treatment option for patients with inflammatory disorders including allergic rhinitis, atopic dermatitis through suppressing MAPK and NF-κB mediated production of inflammatory cytokines.

## Conclusions

Our results clearly indicate that TGT has suppressing activity on expression and production of pro-inflammatory cytokines through inhibition of MAPK and NF-κB signaling pathways in mast cells and keratinocytes. Therefore, TGT can be used to reduce inflammatory symptoms in allergic disorders including not only allergic rhinitis but also atopic dermatitis and asthma.

## Additional files

Additional file 1: Figure S1. Effect of TGT on the viability of PMA and Ionomycin (PI)-stimulated HMC-1 cells. Various concentration of TGT was added to PI treated HMC-1 cells. Cell viability was measured by MTS assay. *; compared to control. #; compared to stimulated cells. Control; HMC-1 with PI (−), Stimulation; HMC-1 with PI (+). (TIFF 82 kb)

Additional file 2: Figure S2. Effect of TGT on the viability of LPS-stimulated HaCaT cells. Various concentration of TGT was added to LPS treated HaCaT cells. Cell viability was measured by MTT assay. *; compared to control. #; compared to stimulated cells. Control; HaCaT with LPS (−), Stimulation; HaCaT with LPS (+). (TIFF 91 kb)

## Abbreviations

ANOVA: One-way analysis of variance
DMEM: Dulbecco’s Modified Eagle’s medium
DMSO: dimethyl sulfoxide
DPBS: Dulbecco’s phosphate-buffered saline
ELISA: enzyme-linked immunosorbent assays
ERK: extracellular signal-regulated kinase;
FBS: fetal bovine serum
GAPDH: glyceraldehyde 3-phosphate dehydrogenase
HMC-1: human mast cell line-1
IgE: immunoglobulin E
IL: Interleukin
IMDM: Iscove’s Modified Dulbecco’s Medium
JNK: c-Jun N-terminal kinase
LPS: Lipopolysaccharide
MAPK: Mitogen activated protein kinase
MTS: 3-(4,5-dimethylthiazol-2-yl)-5-(3-carboxymethoxyphenyl)-2-(4-sulfophenyl)-2H–tetrazolium
MTT: 3-[4,5-dimetylthiazol-2-yl]-2,5-diphenyltetrazoliumbromide
p38: p38 mitogen-activated protein kinase
PBST: Tween-20 phosphate-buffered saline
PI: PMA and ionomycin
PMA: Phorbol 12-myristate 13-acetate
SDS-PAGE: sodium dodecyl sulfate-polyacrylamide gel electrophoresis (SDS-PAGE)
Tc: Cytotoxic T
TGT: Tonggyu-tang
Th: Helper T
TNF-α: Tumor necrosis factor alpha
