# Supplementary figures and images for: Tonggyu-tang, a traditional Korean medicine, suppresses pro-inflammatory cytokine production through inhibition of MAPK and NF-κB activation in human mast cells and keratinocytes
Source: BMC Complement Altern Med. 2017 Mar 31;17:186. doi: 10.1186/s12906-017-1704-5 (PMC5374729; doi:10.1186/s12906-017-1704-5)

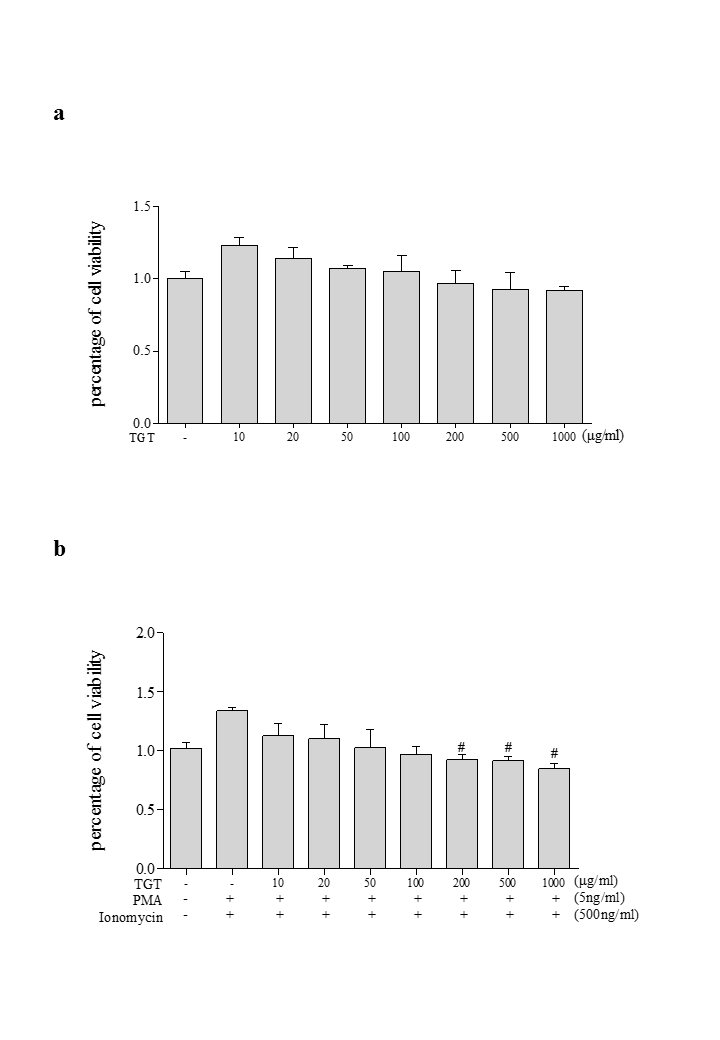

Supplement: Supplementary file 1 — Effect of TGT on the viability of PMA and Ionomycin (PI)-stimulated HMC-1 cells. Various concentration of TGT was added to PI treated HMC-1 cells. Cell viability was measured by MTS assay. *; compared to control. #; compared to stimulated cells. Control; HMC-1 with PI (−), Stimulation; HMC-1 with PI (+). (TIFF 82 kb) [file 12906_2017_1704_MOESM1_ESM.tif]

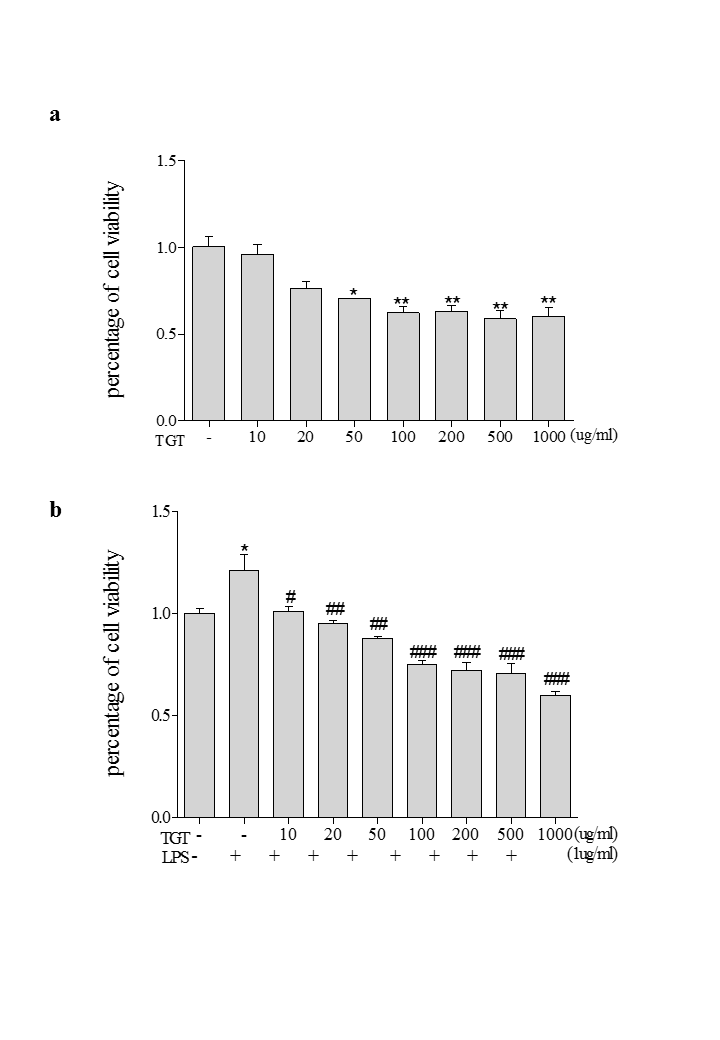

Supplement: Supplementary file 2 — Effect of TGT on the viability of LPS-stimulated HaCaT cells. Various concentration of TGT was added to LPS treated HaCaT cells. Cell viability was measured by MTT assay. *; compared to control. #; compared to stimulated cells. Control; HaCaT with LPS (−), Stimulation; HaCaT with LPS (+). (TIFF 91 kb) [file 12906_2017_1704_MOESM2_ESM.tif]
